# Medical student advising during virtual residency recruitment: results of a national survey of internal medicine clerkship and sub-internship directors

**DOI:** 10.1080/10872981.2022.2143926

**Published:** 2022-11-09

**Authors:** Irene Alexandraki, Nadia Ismail, Cindy J Lai, Nicholas S Duca, Temple Ratcliffe, Michael Kisielewski, Amber T. Pincavage

**Affiliations:** a Department of Academic Affairs, University of Arizona College of Medicine-Phoenix, Phoenix, AZ, USA; b Department of Medicine and Department of Education, Innovation and Technology, Baylor College of Medicine, Houston, TX, USA; c Department of Medicine, University of California at San Francisco, San Francisco, CA, USA; d Department of Medicine, Penn State College of Medicine, Hershey, PA, USA; e Department of Medicine, Joe R. and Teresa Lozano Long School of Medicine, University of Texas Health San Antonio, San Antonio, TX, USA; f Surveys and Research, Alliance for Academic Internal Medicine, Alexandria, VA, USA; g Division of General Internal Medicine, University of Chicago Pritzker School of Medicine, Chicago, IL, USA

**Keywords:** Medical student advising, clerkship, clerkship directors, sub-internship directors, residency interviews, residency application, virtual residency recruitment

## Abstract

**Introduction:**

The residency application process is a critical time for medical students. The COVID-19 pandemic prompted changes to the residency recruitment procedures with the conversion of interviews to a virtual format. For medical school advisors guiding students on an all-virtual residency application process brought uncertainty to their advising practices. Thus, this study aimed to identify advising practices during the 2021 virtual application cycle.

**Methods:**

We administered an IRB-exempt national survey through the Clerkship Directors in Internal Medicine to 186 internal medicine core/co-/associate/assistant clerkship directors and sub-internship directors representing 140 Liaison Committee on Medical Education-accredited U.S./U.S.-territory-based medical schools in spring 2021. The 23-question survey was designed and pilot-tested by faculty-educators and leaders with expertise in undergraduate medical education. Data analysis included paired t- and z-tests and thematic analysis of open-ended questions.

**Results:**

The institutional response rate was 67% (93/140) and individual rate 55% (103/186). Half of the respondents felt prepared/very prepared (40% and 13% respectively) for their advising roles. Compared to pre-pandemic cycles, respondents advised a typical student in the middle-third of their class at their institution to apply to more residency programs (mean 24 programs vs 20, *p* < 0.001) and accept more interviews (mean 14 interviews vs 12, *p* < 0.001). Sixty-three percent (64/101) of respondents spent more time on student advising; 51% (51/101) reported more students asked them for informal advice. Fifty-nine percent (60/101) of respondents reported their advisees were able to assess a residency program ‘somewhat well;’ 31% (31/101) expressed that residency recruitment should remain entirely virtual in the future.

**Conclusion:**

The transition to virtual residency recruitment due to COVID-19 prompted advising practices that may have contributed to application inflation and increased advising workload. Future studies should explore longitudinal outcomes of virtual interviews on student success to guide best practices in how to advise students during residency recruitment.

## Introduction

The residency application process is a stressful time for medical students. Advising can help students navigate the process and maximize their success in the Match [1,2]. The COVID-19 pandemic prompted changes in the residency recruitment processes with the conversion of interviews to a virtual format. These changes raised concerns about the impact of virtual interviews on residency application inflation [3,4], and match success rates [5], and questioned best student advising practices in virtual residency recruitment. Thus, the purpose of this study was to identify advising practices during the 2021 virtual application cycle through a national survey administered by the Clerkship Directors in Internal Medicine (CDIM) and offer a potential reference for medical student advisors.

## Methods

From March to May 2021, we administered an online survey about the virtual IM residency application process to 186 IM core and co-/associate/assistant CDs, and sub-internship directors representing 140 Liaison Committee on Medical Education fully-accredited U.S./U.S.-territory-based medical schools. The 23-question survey was designed, revised after multiple iterations, and pilot-tested by CDIM faculty-educators and leaders with expertise in undergraduate medical education. Eleven of the survey questions focused on student advising for residency (Appendix 1). Data analysis was conducted in *Stata 16.1* and included paired t- and z-tests to compare application cycle outcomes between the pre-2020-21 and the 2020–21 application cycles. For open-ended questions, thematic analysis was conducted following an iterative approach. The study was deemed exempt by Pearl IRB (U.S. DHHS OHRP #IRB00007772).

## Results

The institutional response rate was 67% (93/140) and individual rate 55% (103/186). Almost all respondents (98%, 101/103) served as student advisors for residency. However, only half felt prepared/very prepared (40% and 13% respectively) for their advisor roles.

Compared to pre-pandemicIM residency application cycles, respondents advised a typical student in the middle-third of their class at their institution to apply to more residency programs (mean 24 programs vs 20, *p* < 0.001) and accept more interviews(mean14 vs 12, *p* < 0.001); spent more time advising students (63%, 64/101); and received more requests from students (37%, 31/84) and other individuals (19%, 15/79) to contact residency programs and advocate for students. Half of respondents (51%, 51/101) reported that more students asked for informal advice during the 2020–21 cycle (Table 1).

**Table 1.** Advising practices for internal medicine residency applicants during the virtual application cycle 2020–2021.

Comparing the 2020–21 Virtual Application Cycle with Prior Application Cycles (n = 101)*	Decreased No. (%)	About the same No. (%)	Increased No. (%)
Total number of students advised	4 (4)	75 (74)	22 (22)
Number of students who asked for informal advice	2 (2)	48 (47)	51 (51)
Time spent advising students	0 (–)	37 (37)	64 (63)
Number of students who asked advisor to contact a residency program or advocate on their behalf**	5 (6)	48 (57)	31 (37)
Number of requests from individuals (e.g., other than students) to advocate for a student***	9 (11)	55 (70)	15(19)
Number of residency programs respondents contacted on behalf of student(s)^	5 (6)	52 (63)	25 (31)
**n = 101**	**Pre-2020-2021 application cycle**	**During 2020–2021 application cycle**	
	Mean (SD)	Mean (SD)	*P-value^^*
Number of programs a typical student in the ‘middle-third of their class ’ was advised to apply to	20 (7)	24 (9)	<0.001
Number of interviews a typical student in the ‘middle-third of their class’ was advised to accept	12 (3)	14 (5)	<0.001

SD: standard deviation.

*For 101 respondents who reported to advise students at their institution about internal medicine residency applications. The question asked: ‘How did the following change during the 2020–2021 residency application cycle?’

**Excludes 17 respondents who reported ‘Not applicable:’ n = 84.***Excludes 22 respondents who reported ‘Not applicable:’ n = 79.^Excludes 19 respondents who reported ‘Not applicable:’ n = 82.

^^Paired t-test and paired z-test (using standard deviation of the difference): n = 101.

Fifty-nine percent of respondents (60/101)reported that their advisees could assess a residency program during the virtual interview day ‘somewhat well,’and 9% (9/101) ‘very well.’ From free text responses to a question about challenges their advisees experienced due to virtual residency interviews, 81% (61/75) of respondents felt the virtual interview format made it difficult for students to ‘get a good feel’ of a residency program and its culture, the hospital, and the location. Additionally, 31% (31/101) of respondents expressed that residency recruitment should remain entirely virtual.

## Discussion

The transition to virtual residency recruitment due to the COVID-19 pandemic brought uncertainty and questioned best advising practices. Although, many organizations (e.g., Alliance for Academic Internal Medicine) offered resources for virtual interview preparation for students, advisors, and programs [6,7], our results showed that only half of the respondents felt very prepared/prepared for their roles as student advisors.

During the 2021 virtual residency recruitment, respondents advised a typical student in the middle-third of their class to apply to and interview at a higher number of IM programs which may have contributed to application inflation [3,8,9]. Their advice misaligned with data from the National Residency Matching Program [10] showing that 99% of students would match to IM when their rank list includes 12 or less programs, and that 8–10 interviews would suffice. It is possible that lack of familiarity with these recommendations and uncertainty about the impact of virtual interviews on student candidacy may have been responsible for these findings.

Moreover, advisors received more requests from students and other individuals to contact residency programs, and were asked for informal advice by an increased number of students. One possible explanation may be the ambiguity and angst among students and educators about the virtual residency recruitment process. However, these requests increased advising workload for clerkship leaders raising concerns about burnout [11,12]. Our results underscore the need for institutional support and faculty development interventions to better prepare educators for their advising roles [13].

This study had limitations. Although our respondents were broadly representative of IM clerkships’ advising practices, our findings represent a single specialty and may not be generalizable to other specialties or disciplines. Our survey focused only on advising practices for middle-third ranked students, and variability in student ranking systems among IM clerkships might explain differing responses. Additionally, responses were self-reported which could be subject to perception bias.

In summary, the transition to virtual residency recruitment during COVID-19 prompted advising practices that may have contributed to higher application numbers and increased advising workload. Future studies should explore longitudinal outcomes of virtual interviews.

## Data Availability

The data that support the findings of this study are openly available in Harvard Dataverse (‘Replication Data for: 2021 AAIM/CDIM Survey on the Virtual Residency Application Process’) at https://doi.org/10.7910/DVN/Y05ULM, reference number UNF:6:7hhL0iDwfeOXP4HAyviCBw== [fileUNF].

## Appendix 1

### 2021 CDIM Spring Survey of Internal Medicine Clerkship and Sub-Internship Directors

**Start of Block: Landing Page**

**2021 CDIM Spring Survey on the Virtual Residency Application Process**

The purpose of this survey is to 1. understand the perspectives of internal medicine (IM) clerkship and sub-internship directors (as student advisors) about the 2020–21 residency application cycle when interviews were conducted virtually; 2. identify perceptions of how your advising might have changed; and 3. gather data essential for providing medical schools and residency programs with recommendations about the virtual application process.

The survey results will be compared to the 2021 APDIM Spring Survey of Residency Program Directors on Virtual Interviewing, to provide medical educators with more holistic information. Summary results from both surveys will be available at IM.org in the months after survey closure. **Upon completing this survey, you will receive your responses by email.**

**The survey should take approximately 15 minutes to complete**. At any point, you may exit and return without losing your data. Please use the unique survey link in your email invitation; you will be returned to where you left off. The survey software will alert you of any unanswered questions but you may skip any that you do not wish to answer.

This study (#21-AAIM-118) is exempt by Pearl IRB (U.S. DHHS OHRP #IRB00007772) under FDA 21 CFR 56.104 and 45 CFR46.104(b)(2). You are invited to participate as an IM core clerkship or sub-internship director whose institution is a CDIM member: co-/associate/assistant clerkship directors are included. **Participation is voluntary**; refusal to participate will not affect your/your institution’s membership. Upon survey closure, all personal and institutional identifiers will be removed by Alliance for Academic Internal Medicine Surveys staff, who manage data collection.

If you encounter technical problems, no longer are a clerkship or sub-internship director, or have questions about the survey content, contact surveys@im.org or 703–341-4540. If you feel that your participant rights have not been upheld, please contact Pearl IRB at info@pearlirb.com or 317–602-5917:

****SURVEY NAVIGATION****

1. **DO NOT USE your browser’s ‘Back’ or ‘Forward’ buttons to navigate the survey**. Please use the survey <PREVIOUS> and <NEXT> buttons at the bottom of each page.

2. This survey is compatible with most tablet devices but if you encounter technical problems please check that your device’s operating system is updated. Smartphones use is discouraged, due to programming that might cause unexpected survey navigation problems. Further **technical support FAQ’s** may be viewed here (a separate browser window/tab will open).

Q1 ***By clicking below, you acknowledge that your participation is voluntary.***

▢ *Click ‘PROCEED’ (below) to begin*

*Display This Question:*

*If Q1 ! = Click ‘PROCEED’ (below) to begin*

Q2 ***TO CONFIRM: Do you acknowledge that your participation is voluntary?***

▢ ***Yes: BEGIN the survey***
▢ ***No (you will EXIT the survey and not be able to return)***
▢

*Skip To: End of Survey If Q2 = No (you will EXIT the survey and not be able to return)*

**End of Block: Landing Page**

**Start of Block: Section I: Student Advising About IM Residency Applications**

**Student Advising for Internal Medicine Residency Applications**

Q3 **Do you advise students at your institution about internal medicine residency applications? *Answer carefully; your response will be used for subsequent questions.***

o No
o Yes

*Skip To: End of Block If Q3 = No*

*Display This Question:*

*If Q3 = Yes*

Q4 ***For the periods below, enter whole numbers only.***

***On average*, for how many of the following did you advise a typical ‘middle-third-of-the-class’ student at your institution?**

**Table:**

	… Number of PROGRAMS to APPLY to?	… Number of INTERVIEWS to ACCEPT?
***PRIOR to*** the 2020–2021 application cycle (pre-COVID-19)		
***DURING*** the 2020–2021 application cycle (COVID-19)		

**End of Block: Section I: Student Advising About IM Residency Applications**

**Start of Block: Section II: IM CDs and Sub-I Directors’ Perceptions of their Advising Practices**

*Display This:*

*If Q3 = Yes*

**Perceptions of Your Advising Practices and Residency Application Process Before and During the Pandemic**

*Display This Question:*

*If Q3 = Yes*

Q5 ***Please answer for the pandemic application cycle (2020–2021), compared to PRIOR cycles (pre-COVID-19)*. How did the following change during the 2020–2021 residency application cycle?**

**Table:**

	Decreased	About the same	Increased	Not Applicable	Unsure
Total number of students you advised	o	o	o	o	o
Number of students who asked you for informal advice	o	o	o	o	o
Your time spent advising students	o	o	o	o	o
Number of students who asked you to contact a residency program or advocate on their behalf	o	o	o	o	o
Number of requests you received from individuals (e.g., other than students) to advocate for a student	o	o	o	o	o
Number of residency programs you contacted on behalf of student(s)	o	o	o	o	o

*Display This Question:*

*If Q3 = Yes*

Q6 **How prepared were you for your advisor’s role during the 2020–2021 residency application cycle when interviews were conducted virtually?**

o Very unprepared
o Unprepared
o Neutral
o Prepared
o Very prepared
o Do not know/Unsure

*Display This Question:*

*If Q3 = Yes*

Q7 ***Answer to the best of your ability*: Compared with *pre-COVID-19* application cycles, how likely were your current advisees to …**

**Table:**

	Less likely	Neither less likely nor more likely	More likely	Do not know/Unsure
Receive invitations for out-of-state interviews?	o	o	o	o
Match at your institution’s residency program?	o	o	o	o
Match at regional residency programs?	o	o	o	o

*Display This Question:*

*If Q3 = Yes*

Q8 **Generally speaking, how well could your current advisees assess a residency program(s) during the virtual interview day?**

o Not at all well
o Somewhat well
o Very well
o Other (please explain): _________
o Do not know/Unsure

*Display This Question:*

*If Q3 = Yes*

Q9 **If onsite interviews are an option, do you think that the residency recruitment process should remain entirely virtual next year?**

o No
o Yes
o Other (please explain): _________

*Display This Question:*

*If Q3 = Yes*

Q10 **If unrestricted travel is allowed next year, what aspect(s) of virtual interviewing would you like to retain for the 2021–2022 residency interview season? *Check all that apply.***

▢ Option for virtual interviews with faculty (some or all)
▢ Option for virtual interviews with residents
▢ Option for a full virtual interview cycle
▢ Other (please explain): _________
▢ ⊗Do not know/Unsure
▢ ⊗None of the above

*Display This Question:*

*If Q3 = Yes*

Q11 **What challenges did your students experience due to virtual residency interviews? *Answer to the best of your ability.*** ____________

**End of Block: Section II: IM CDs and Sub-I Directors’ Perceptions of their Advising Practices**
